# Adequacy of Operative Notes for Orthopaedic Procedures: A Quality Improvement Project

**DOI:** 10.7759/cureus.96179

**Published:** 2025-11-05

**Authors:** Atizaz A Jan, Hassan Imtiaz, Talha Ahmed, Omran Alkhatib, Adam Khan Rahim, Georgios Kouklidis

**Affiliations:** 1 Trauma and Orthopaedics, University Hospitals Dorset NHS Foundation Trust, Poole Hospital, Poole, GBR; 2 Internal Medicine, Hayatabad Medical Complex Peshawar, Peshawar, PAK; 3 Orthopaedics, University Hospitals Dorset NHS Foundation Trust, Poole Hospital, Poole, GBR

**Keywords:** clinical audit, clinical governance, operation notes, orthopaedics surgery, quality improvement (qi)

## Abstract

Introduction: High-quality operative documentation is fundamental to safe patient care, supporting communication, guiding postoperative management, and serving as a medico-legal record. Despite its importance, variability in the quality and completeness of operative notes remains common.

Methods: A closed-loop audit was performed at University Hospital Crosshouse, United Kingdom. The first cycle reviewed 82 operative notes, assessing compliance with set standards. Interventions included encouragement to use typed instead of written operation notes, display of posters in theatres, and introduction of a structured post-operative checklist. A second cycle reviewed 84 notes post-intervention. Results between audit cycles were compared and analysed using chi-squared tests to evaluate the statistical significance of improvements.

Results: In Cycle 1, typed notes accounted for 25 (30%), with documentation of operative diagnosis in 53 (65%), operative findings in 60 (73%), closure technique in 73 (89%), and signature completion in 78 (95%). Estimated blood loss was recorded in only five (6%) notes, and prosthesis identification (where applicable) in 60 of 69 (87%). In Cycle 2, following the intervention, typed notes increased significantly to 55 (65%). Closure technique documentation improved to 84 (100%) and signature completion to 84 (100%). Modest improvements were found in documentation of operative diagnosis (n=62, 74%) and intra-operative findings (n=68, 81%). Prosthesis identification remained at 87% between audit cycles, and estimated blood loss documentation fell to 2 (2%).

Conclusion: Simple, low-cost interventions such as visible documentation standards, typed templates, and postoperative checklists produced meaningful improvements in legibility and completion of key operative note parameters. Sustaining and expanding these measures, particularly through electronic templates with mandatory data fields and ongoing audit cycles, could further enhance the accuracy, completeness, and medicolegal robustness of operative documentation within surgical practice

## Introduction

High-quality operative documentation underpins surgical practice. Operative notes ensure continuity of care, guide postoperative management, and serve as vital medico-legal records. Deficient documentation has been linked to communication failures, adverse outcomes, and litigation risk [1,2].

The General Medical Council (GMC), through Good Medical Practice, requires doctors to record work clearly, accurately, and contemporaneously [3]. The Royal College of Surgeons of England (RCS Eng) builds on this with Good Surgical Practice, outlining a checklist of essential operative note items, including operative diagnosis, complications, prophylaxis, tissue removed, prostheses, and closure technique [4]. These standards provide an evidence-based framework to reduce omissions and variability.

Despite clear guidance, audits across surgical specialties consistently demonstrate incomplete and inconsistent operative notes [5-7]. Barriers include time pressures, reliance on freehand documentation, lack of awareness of standards, and absence of structured templates [8,9]. To address these, quality improvement measures, such as standardised proformas, electronic note systems, and checklists, have been shown to significantly improve compliance [10-13].

Given the central role of documentation in safe care, regular auditing is recommended [14]. We report a closed-loop audit assessing orthopaedic operative notes against GMC and RCS Eng standards, evaluating the impact of targeted interventions.

## Materials and methods

This prospective closed-loop audit was undertaken at the Trauma & Orthopaedics department, University Hospital Crosshouse, NHS Ayrshire and Arran. The first cycle was conducted between 01/09/2024 and 15/09/2024, while the subsequent cycle was conducted between 15/10/2024 and 30/10/2024. Following the first cycle, gaps were identified in practice. To address these, posters (Appendix 1) highlighting key details to be documented on operative notes were displayed in operating theatres. Additionally, a post-operative checklist was introduced, which was mandatory to be completed prior to the patient leaving the theatre recovery (Appendix 2), with the aim to ensure that important post-operative instructions are not missed when a patient leaves of theatre. The orthopaedic team was encouraged to rely more on typed rather than hand-written operative notes, and preferably use templated notes (if available), which cover all relevant aspects of the procedure, to ensure better documentation.

Consecutive operative notes were analysed for the given timeframes. Data were collected from Electronic Patient Records (EPR) and inpatient paper records for assessment of operation notes. All orthopaedic surgery cases (including both emergency and elective procedures) were evaluated during the specified time frames for each audit cycle. Surgeries done under local anesthetic or non-orthopaedic surgeries were excluded.

Audit standards were derived from the GMC's guidance on Good Medical Practice [3] and the RCS's Good Surgical Practice [4] guidelines for documentation on operative notes. Based on these guidelines, the documentation of the following parameters was evaluated for each operative note: date and time, elective or trauma procedure, name of operating surgeon with assistant and anesthetist, name of procedure, indication for procedure/diagnosis, intra-operative findings, identification of prosthesis used (where applicable), estimated blood loss, post-operative instructions including follow-up plans, and signature by operating surgeon. Target compliance to documentation for each parameter was mutually agreed among orthopaedic team members and set at 100%.

Data were collected and stored in Microsoft Excel (Microsoft® Corp., Redmond, WA), and analysed using JASP software (version 0.18.3). Patient identifiers were removed from the datasheet prior to final analysis. Chi-square test was employed to statistically compare the results of the first and second loop results, with a p-value of less than 0.05 being set as a mark of significance.

## Results

A total of 82 operative notes were reviewed during the first audit cycle. Documentation was complete for key identifiers, such as the date, name of surgeon, assistant, anaesthetist, and procedure, in 100% of cases. Typed notes accounted for 25 (30%) of all entries, with the majority remaining handwritten. Operative diagnosis was documented in 53 notes (65%), while operative findings were recorded in 60 (73%). Among the 69 cases involving prosthesis use, identification details were specified in 60 (87%). Estimated blood loss was documented in only five notes (6%). Closure technique was described in 73 (89%) cases, detailed postoperative instructions were provided in 78 (95%), and the surgeon's signature was present in 78 (95%) of the notes. Documentation of time and whether the procedure was elective or trauma-related remained suboptimal at 12% and 27%, respectively. Overall, while core identifying information was consistently recorded, several key clinical and procedural elements demonstrated room for improvement, particularly in operative findings, prosthesis identification, and estimated blood loss.

Following the implementation of interventions, including a theatre poster displaying the documentation standards, encouragement to use typed operation note templates, and introduction of a post-operative checklist, 84 operation notes were assessed in the second audit cycle. Typed documentation increased markedly to 55 notes (65%), representing a statistically significant improvement compared with Cycle 1 (p<0.001). Closure technique documentation improved to 100% (84/84), also showing a significant increase (p=0.038). Similarly, signature completion rose to 100% (p=0.040). Operative diagnosis and findings improved modestly to 74% and 81%, respectively, though these changes were not statistically significant (p>0.05). Prosthesis identification among the 54 applicable cases remained stable at 87%. Estimated blood loss documentation fell slightly to 2% (p=0.19), while postoperative instruction documentation increased marginally to 98% (p=0.39). The date, surgeon details, and procedure name continued to achieve 100% compliance in both cycles. Overall, the second audit cycle demonstrated substantial gains in legibility through typed notes and in completeness for closure technique and signature, while other parameters showed positive but non-significant improvements. The results of both audit cycles, along with statistical analysis, are presented in Table 1.

**Table 1:** Comparison between first and second audit cycles, with statistical analysis * Prosthesis usage: 69 cases in the first audit cycle, 54 cases in second audit cycle. ** p-value of less than 0.05 considered statistically significant

Parameter	Cycle 1 (n=82)	Cycle 2 (n=84)	Change (%)	Chi-squared value(χ²)	p-value**	Statistically Significant(Yes/No)
Typed notes	25 (30%)	55 (65%)	+35	20.345	<0.001	Yes
Date	82 (100%)	84 (100%)	0	-	-	-
Time	10 (12%)	9 (11%)	−1	0.090	0.764	No
Trauma or elective procedure	22 (27%)	20 (24%)	−3	0.200	0.655	No
Name of surgeon, assistant, and anaesthetist	82 (100%)	84 (100%)	0	-	-	-
Operative diagnosis	53 (65%)	62 (74%)	+9	1.388	0.239	No
Name of Procedure	82 (100%)	84 (100%)	0	-	-	-
Operative findings	60 (73%)	68 (81%)	+8	1.068	0.301	No
Prosthesis identification*	60/69 (87%)	47/54 (87%)	0	0.000	1.000	No
Estimated blood loss	5 (6%)	2 (2%)	−4	1.693	0.193	No
Closure technique	73 (89%)	84 (100%)	+11	4.291	0.038	Yes
Detailed post-operative instructions	78 (95%)	82 (98%)	+3	0.743	0.389	No
Signature	78 (95%)	84 (100%)	+5	4.199	0.040	Yes

## Discussion

This closed-loop audit demonstrates that straightforward, low-resource interventions can produce measurable improvements in the quality of operative documentation in orthopaedic practice. The most striking change was the shift from handwritten to typed notes (30%-65%), a statistically significant improvement. This is consistent with prior work showing that typed or electronic operation notes improve legibility and completeness and reduce missing data compared with freehand notes [11,12]. The conversion to typed notes likely addresses two common root problems: poor legibility and potential omissions due to time pressure or handwriting deterioration. It has the secondary benefit of facilitating future integration into electronic proformas and mandatory fields that prompt clinicians.

Documentation of the closure technique and signature also improved significantly after the intervention. Both of these items are critical for postoperative care, accountability, and medicolegal safety; similar gains in these domains have been reported after the introduction of checklists and proformas in other surgical settings [1,10]. The mandatory postoperative checklist used in our intervention likely reinforced completion of closure and signatory fields prior to leaving recovery, explaining the observed improvement.

Several other domains showed improvement that did not reach statistical significance: operative diagnosis, operative findings, and detailed postoperative instructions. Prosthesis identification (87% in both cycles) remained high and unchanged when assessed among the subset of cases where a prosthesis was used. These findings align with the pattern reported in systematic reviews and audits that find the largest improvements occur in items that are explicitly prompted by templates or checklists, whereas more narrative items (findings, diagnosis detail) improve less unless templates include dedicated prompts or mandatory fields [13,15].

Estimated blood loss documentation decreased, but this change was not statistically significant (p=0.193). Low baseline rates of documentation for such parameters can reflect variability in perceived clinical relevance for routine orthopaedic procedures; embedding these fields into electronic templates has been shown elsewhere to increase capture rates [16].

Our results mirror prior literature showing the effectiveness of simple interventions (proformas, checklists, education, and electronic templates) in improving operation note quality [1,10,15,16]. The audit also highlights an important practical point: items already at or near 100% compliance (e.g., date, surgeon name, procedure name) are less amenable to further change, and efforts may be better directed at items with lower baseline capture or those that will impact patient safety. Introducing typed or electronic, templated notes with mandatory fields for key safety items is a logical next step and is supported by health informatics studies showing better compliance and legibility with electronic operation notes [17].

Limitations include the single-centre nature of the audit and modest sample sizes, which reduce power to detect small differences for parameters with intermediate baseline compliance. Additionally, the time frame between audit cycles was only a month, and it is possible that a much delayed re-audit could have resulted in larger improvements and produced a more reliable indication of the sustainability of implemented changes following the first audit cycle.

## Conclusions

This audit demonstrates that simple, low-cost interventions can lead to meaningful improvements in the quality of operative documentation. The introduction of theatre posters, a typed template, and a postoperative checklist significantly increased the proportion of typed notes and achieved full compliance in closure technique and signature documentation. These results confirm that targeted educational and process-based measures can effectively enhance adherence to GMC and RCS documentation standards, improving both legibility and completeness of operative notes.

Although some parameters, such as operative findings and estimated blood loss, showed limited or no improvement, the overall trend indicates greater consistency and awareness among operating surgeons. Sustained improvement will likely depend on embedding these interventions into routine practice. Future work should focus on implementing electronic operation note systems with mandatory data fields, regular re-audits, and feedback to ensure continued compliance and standardisation. By promoting structured, legible, and comprehensive documentation, such measures will strengthen patient safety, clinical communication, and medico-legal protection within surgical practice.

## Figures and Tables

**Figure 1:**
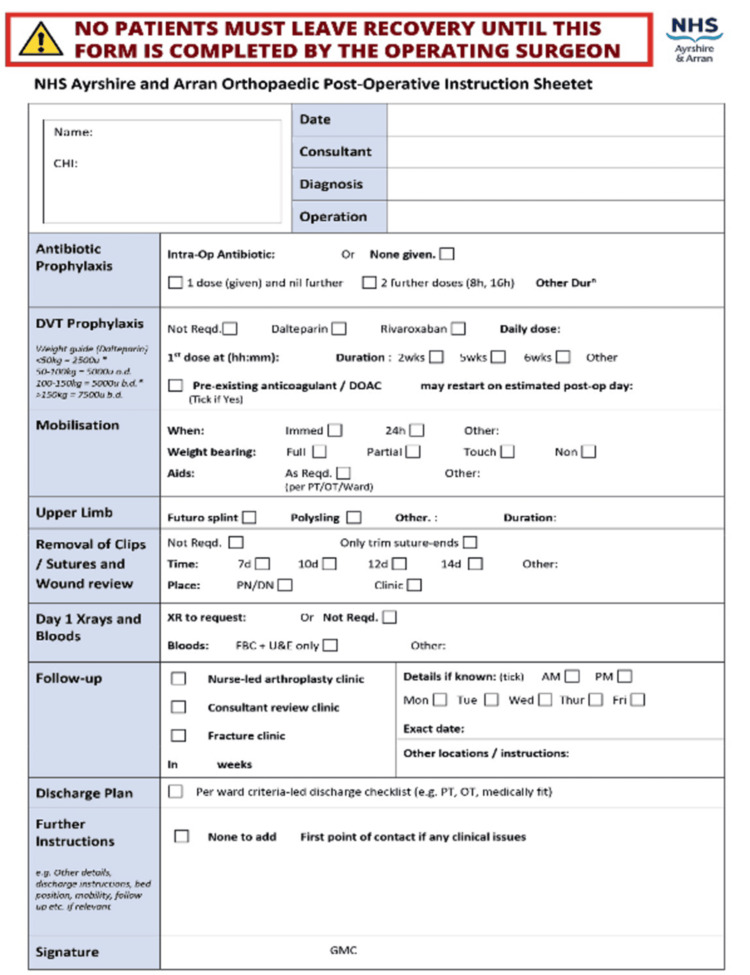
Appendix 2 (Post-operative recovery checklist) The post-operative checklist was introduced, which was mandatory to be completed prior to the patient leaving the theatre recovery.

## Appendices

**Table 2:** Appendix 1 (Poster displayed in theatres) Poster highlighting key details to be documented on orthopaedic operative notes were displayed in operating theatres to serve as reminders.

RCos Guidance on Parameters to be included in Operative Notes	Tick if completed
Operative notes typed up (preferable)	
Date of procedure	
Time of procedure	
Elective or emergency procedure	
Name of operating surgeon and assistant	
Name of theatre anaesthetist	
Operative procedure carried out	
Incision	
Operative diagnosis	
Operative findings	
Any problems/complications	
Any extra procedure performed and why	
Details of tissue removed, added, or altered	
Identification of any prosthesis used, including serial numbers of prosthesis and other implanted materials	
Details of the closure technique	
Anticipated blood loss	
Antibiotic prophylaxis (where applicable)	
DVT prophylaxis (where applicable)	
Detailed post-operative care instructions	
Signature	
Follow-up	
